# Hospital-wide pathogen transmission surveillance combining epidemiological, genetic, and spectroscopic approaches

**DOI:** 10.1128/spectrum.02230-25

**Published:** 2026-05-26

**Authors:** Sophia Wolf, Elisabeth Barth-Jakschic, Baris Bader, Silke Peter, Jan Liese

**Affiliations:** 1 Institute of Medical Microbiology and Hygiene, University Hospital Tübingen 199902, Tübingen, Germany; 2 German Center for Infection Research (DZIF), Partner Site Tübingen 459706, Tübingen, Germany; University of Heidelberg, Heidelberg, Germany

**Keywords:** nosocomial infections, outbreak detection, pathogen typing, whole-genome sequencing, Fourier-transform infrared spectroscopy

## Abstract

Nosocomial infections, particularly those caused by multidrug-resistant organisms, pose a significant challenge in healthcare facilities. This study presents a novel approach that combines tracking patient contacts in the hospital with Fourier-transform infrared (FTIR) spectroscopy and whole-genome sequencing (WGS) of bacterial isolates for rapid surveillance of nosocomial pathogen transmissions. Isolates from relevant species were collected over a 2-month period, and FTIR spectroscopy and WGS were performed on strains suspected in transmission events. The integrated approach identified 94 suspected transmission events involving 103 patients, with single nucleotide polymorphism analysis confirming 32% of these transmissions. FTIR spectroscopy demonstrated good specificity and moderate sensitivity compared to WGS, with the performance highly dependent on the bacterial species analyzed. Room contact of the patients played an important role in pathogen transmission, particularly with *Klebsiella pneumoniae*. This described approach offers a rapid and accurate means of identifying and tracking the spread of nosocomial pathogens, thereby enhancing infection control strategies and improving patient safety.

**IMPORTANCE:**

This manuscript presents a study that employs pathogen typing and monitors hospital occupancy data for the detection of transmissions of hospital-acquired pathogens. The results demonstrate that this approach can be a powerful tool for real-time surveillance and control of nosocomial infections in healthcare settings. The findings have important implications for improving patient safety and reducing the spread of multidrug-resistant organisms in hospitals.

## INTRODUCTION

Hospital-acquired (nosocomial) infections are a major problem in healthcare settings worldwide and can be caused by a variety of bacterial, viral, and fungal pathogens (1, 2). Infections caused by multidrug-resistant organisms (MDROs) are of special concern due to very limited treatment options and the rapid spread of these pathogens within and between hospitals (3, 4). Transmission of pathogens can occur through various means, including direct contact with infected individuals or contaminated surfaces, and can lead to outbreaks within hospitals, especially if the acquisition of the causing microorganism by the patients remains undetected for a long time (5, 6). Nosocomial infections and outbreaks are associated with high morbidity, mortality, and increased costs; therefore, rapid detection of pathogen transmission in the hospital is crucial in preventing their spread and controlling outbreaks (1). However, it is sometimes difficult to determine the extent of an outbreak if common bacterial species without distinctive features are involved or if clinical samples are not available. The accurate determination of transmission relies on the establishment of clonal relationships between bacterial isolates and detecting epidemiological links between patients, which usually requires processing of large data sets containing microbiological and hospital occupancy data. Automated or semi-automated methods have been described for outbreak detection to facilitate this task (7, 8). However, these surveillance methods often focus on MDROs because of the described relevance for hospitalized patients, although the majority of nosocomial infections are caused by antibiotic-sensitive strains in many settings (9), which can delay the detection of transmission chains caused by these pathogens even further.

The results of routine bacterial identification and susceptibility testing in the clinical microbiology laboratory usually do not provide the required granularity for strain typing, which would be needed for the detection of clonal strains in different patients. As a result, additional methods for rapid and accurate identification of pathogens and for determining the relatedness of isolates are needed, such as multi-locus sequence typing (MLST) or pulse-field gel electrophoresis. Another technique that has been employed for these purposes is Fourier-transform infrared (FTIR) spectroscopy, which has shown promising results for bacterial identification and typing and therefore represents a potentially useful approach for the detection of transmission events (10–12). This method determines the absorption of infrared light by chemical bonds present in a sample to analyze its composition and can be used in the clinical microbiology laboratory to examine bacterial cells that form colonies on agar plates. FTIR enables rapid species identification and has also been evaluated as a fast and cost-effective tool for strain typing. In several studies, FTIR-based clustering of gram-negative bacilli in hospital outbreaks showed good concordance with molecular typing methods such as MLST or whole-genome sequencing (WGS). The strongest performance and most extensive experience have been reported for *Klebsiella pneumoniae* (11, 13–15), whereas results for *Enterobacter cloacae* complex and *Pseudomonas aeruginosa* have been more variable, reflecting species-dependent differences in FTIR discriminatory power (12, 16). This variability should be considered when interpreting clustering results and planning outbreak or transmission surveillance (10–12, 16). On the other hand, whole-genome sequencing has emerged as a powerful tool for tracking nosocomial pathogens and has been widely used to identify transmission events within hospitals or in the community (17). Investigating WGS data by single nucleotide polymorphism (SNP) analysis or whole-genome MLST is currently becoming the gold standard for bacterial typing (18).

Here, we present a study that combines FTIR spectroscopy and WGS for transmission surveillance in a hospital setting. Clinical microbiology results and hospital occupancy records were continuously scanned for possible transmission events, and the implicated bacterial isolates were further investigated by both methods. The aims were to determine the performance of such a cluster detection system for different species in the hospital and to assess the usefulness of FTIR spectroscopy in detecting these events.

## MATERIALS AND METHODS

### Strain collection and bacteriological procedures

Bacterial isolates (*n* = 1,543) of the species *Staphylococcus aureus*, *Pseudomonas aeruginosa*, *Acinetobacter baumannii* complex, *Stenotrophomonas maltophilia*, and *Enterobacterales* (except for *Escherichia coli*) were collected during a 2-month study period (November 2019 until January 2020) from the routine diagnostic microbiological laboratory. Clinical and screening isolates were included in the study. One isolate per patient (preferably the first isolate) was collected for each species. Species identification was performed by linear matrix-assisted laser desorption/ionization time-of-flight mass spectrometry (MALDI-TOF MS) on a MALDI Biotyper system (based on a Microflex LT/SH instrument; Bruker Daltonik, Bremen, Germany). Isolates were obtained from purity control plates (Columbia sheep blood agar plates; Oxoid, Wesel, Germany), plated on Müller-Hinton agar (Oxoid, Wesel, Germany), and stored at 4°C. Isolates involved in suspected transmission events (see below) were recovered on Columbia sheep blood agar plates for spectroscopic and genomic analyses and finally stored at −80°C.

### Screening of hospital occupancy data for potential transmissions

**Fig 1:**
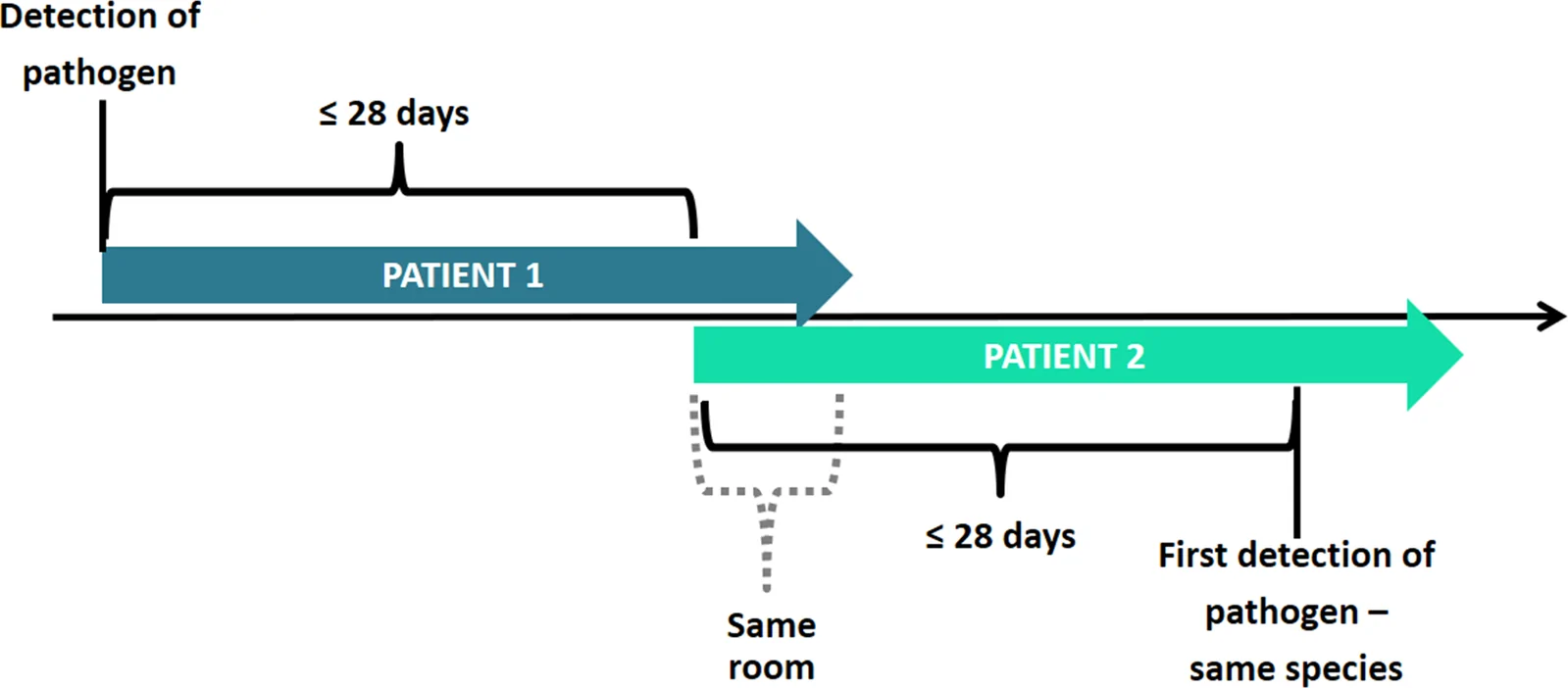
Definition of potential patient-to-patient transmission. Patient-to-patient transmission was suspected if two patients were co-located in the same room for at least one night (dashed bracket) and the respective pathogen species was found in samples from both patients. The pathogen had to be detected from samples of the “donor” (patient 1) within 4 weeks before the room contact and from specimens of the “recipient” (patient 2) within 4 weeks after the first contact.

A database was constructed that contained information about the location of every patient on every day at midnight (date, ward number, and room number) and microbiological diagnostic results (date of specimen collection and bacterial species). Both tables were connected by the patient’s ID and updated every workday. After updating, an algorithm identified potential transmissions as defined in Fig. 1. Both bacterial isolates in newly detected potential transmissions were retrieved from the strain collection and subjected to further analysis.

### Whole-genome sequencing

Bacterial DNA was extracted using the DNeasy Ultraclean Microbial Kit (Qiagen, Venlo, the Netherlands). DNA libraries were prepared with the TruSeq DNA HT Sample Prep Kit (Illumina, San Diego, CA, USA). Normalized libraries were pooled and sequenced with a 300 Cycle Mid-Output Cartridge on a NextSeq platform (Illumina, San Diego, CA, USA).

### Assembly and genome analysis

The assembly of sequence reads was performed using the A5 pipeline (version 20140604) and SPAdes (version 3.7.0; 19, 20). Trimmomatic was used to generate high-quality reads (21). Core genomes were generated using Spine (version 0.1.2) (22). Prophage regions were determined and removed using PHASTER_2_ (23). Core-genome-based SNP calling was performed by mapping high-quality reads to the core genome using Bionumerics 7.6 (Applied Maths, Sint-Martens-Latem, Belgium) with default settings. Multi-locus sequence types (STs) were extracted from the assembled genomes using the online service by the Center for Genomic Epidemiology (version 2.0) based on the MLST scheme of the respective species (24). For species identification, the average nucleotide identity was calculated based on the ANIm algorithm using JSpecies (version 1.2) (25).

For *S. aureus*, *spa* types were predicted from the assembled genomes using the online service provided by the Center of Genomic Epidemiology (26). Capsule types of *K. pneumoniae* were determined using the online tool Kaptive (27).

### FTIR spectrum acquisition and analysis

Strains were plated on Columbia sheep blood agar plates (Oxoid, Wesel, Germany) and incubated at 37°C for 24 h (±30 min). To suspend the bacteria, one loop full of bacteria was added to 50 µL of 70% (vol/vol) ethanol in 1.5 mL vials and thoroughly vortexed. After adding 50 µL of sterile H_2_O, 15 µL of the bacterial suspension was placed on a silicon sample plate (Bruker Daltonik, Bremen, Germany), and the sample plate was dried at 37°C for approximately 20 min.

The measurements were performed using an IR Biotyper System (Bruker Daltonik, Bremen, Germany) running the IR Biotyper software (version 1.5) with the default settings. Measurements that did not meet the default quality criteria (absorption, 0.4–2.5; noise [×10^−6^], <250; signal-to-noise ratio, >40; and water vapor [×10^−6^], <300) were excluded from further analysis. Spectrum processing included calculating the second derivative of the 1,300–800 cm^−1^ wavenumber range of the spectra, vector normalization, and calculation of respective summary spectra. For each isolate, four technical replicates were generated in each of three independent experiments, resulting in three biological isolates, which were summarized to one isolate spectrum. Based on the Euclidean distance between the isolate spectra, clustering was performed with the BioNumerics 7.6 software suite (Applied Maths, Sint-Martens-Latem, Belgium) using the UPGMA algorithm. The relatedness between isolates is expressed as similarity values in percentage based on the Euclidean distance and displayed in the corresponding figures.

## RESULTS

### Transmission surveillance based on hospital occupancy and microbiology data

During a 2-month study period, all bacterial isolates of the target species *Staphylococcus aureus*, *Pseudomonas aeruginosa*, *Acinetobacter baumannii* complex, *Stenotrophomonas maltophilia,* and several species within the order of *Enterobacterales* (except for *Escherichia coli*) were collected from the routine diagnostic microbiological laboratory. Clinical and screening isolates were included, and one isolate per patient (preferably the first isolate) of the respective species was stored at 4°C. This resulted in a collection of 1,543 strains (Table 1).

**TABLE 1:** Species distribution of collected isolates in relation to potential and confirmed transmissions for each species^*a*^

Species	Total number of isolates collected	Patients/isolates involved in potential transmissions (percentage of total isolates)	Number of potential transmissions	Confirmed transmissions by SNP analysis	Transmissions detected by FTIR spectroscopy	
					Correct	Incorrect
*K. pneumoniae*	138	12 (8.7%)	17	13	10	0
*E. cloacae* complex	118	24 (20.3%)	30	15	13	0
*S. aureus*	427	35 (8.2%)	28	2	1	4
*P. aeruginosa*	369	26 (7.0%)	14	0	0	0
Other *Enterobacterales*	444	0 (0.0%)	0	nd	nd	nd
*A. baumannii* complex	17	4 (23.5%)	4	nd	nd	nd
*S. maltophilia*	30	2 (6.7%)	1	nd	nd	nd
Total	1,543	103 (6.7%)	94	30	24	4

^*a*^ nd, not determined.

At the same time, hospital occupancy data and diagnostic microbiological reports were perpetually screened by an electronic algorithm for potential patient-to-patient transmissions of pathogens. Transmission was suspected if two patients were co-located in the same room for at least one night and if the same bacterial species was detected within 4 weeks before room contact in samples from one patient (donor) and within 4 weeks after the first contact in samples from the other patient (recipient) (Fig. 1). All patients (and their respective bacterial isolates) that were connected by suspected transmission events were grouped in “patient clusters.”

This epidemiological surveillance revealed a total of 94 suspected transmissions involving 103 patients (note that one patient could be involved in more than one transmission event). *S. aureus* and *P. aeruginosa* were the most frequently found pathogens in clinical specimens, but they were part of only 28 and 14 suspected transmissions, respectively. The rate of isolates involved in suspected transmissions was the highest for *E. cloacae* complex (Table 1). Only a very few suspected transmissions were detected for *Enterobacterales* other than *K. pneumoniae* and *E. cloacae* complex, as well as involving *A. baumannii* complex and *S. maltophilia* (Table 1). Therefore, these pathogens were omitted from further analysis.

Subsequently, all isolates from presumed transmission events were subjected to WGS. SNP analysis was then performed to determine if isolate pairs had a clonal relationship, which would confirm the transmission. Genetically related isolates were grouped in “SNP clusters,” which were defined using species-specific pairwise SNP thresholds (28).

These isolates were also analyzed by FTIR spectroscopy to assess the accuracy of this fast and easy-to-perform typing technique in this surveillance setting. If two or more isolates of one species exhibited a spectral similarity above a species-specific threshold, these isolates were grouped in an “FTIR cluster.”

Species-specific thresholds have been defined previously for *K. pneumoniae* (11) and *E. cloacae* complex (12). In the absence of established cutoff values for *S. aureus* and *P. aeruginosa*, thresholds guided by those defined for the other species were used.

### Transmission surveillance and typing of *K. pneumoniae*

**Fig 2:**
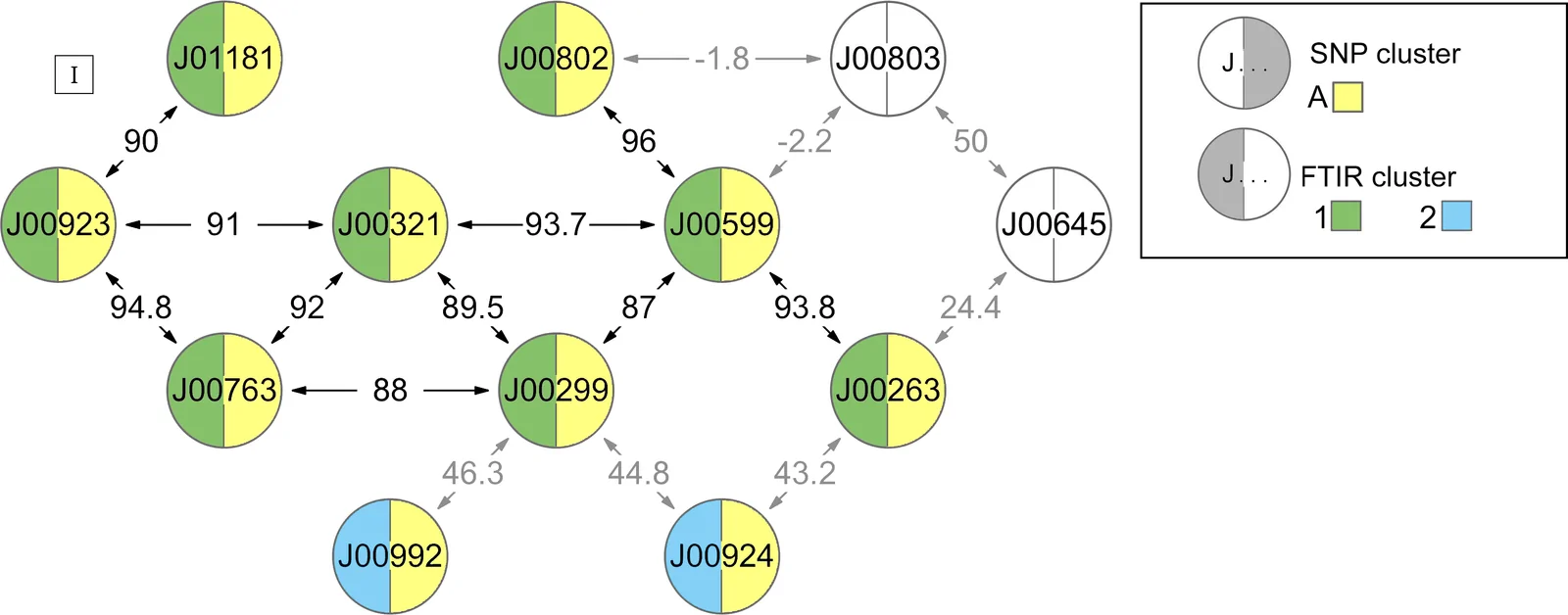
Transmission surveillance of *K. pneumoniae*. For *K. pneumoniae,* 17 potential transmissions (black and gray arrows) among 12 patients/isolates (circles with isolate ID) in 1 cluster (Roman numeral) were suspected based on epidemiological data. WGS revealed one SNP cluster (colored right half of the circles; cutoff value: 18 SNPs) involving 10 isolates and 2 singletons (uncolored). FTIR typing revealed two FTIR clusters (colored left half of the circles) and two singletons (uncolored). Arrows and numbers indicate the FTIR spectrum similarities between isolates above (black) and below (gray) the cutoff value (75%).

Among the 138 collected *K. pneumoniae* strains, 12 patient isolates were involved in 17 potential transmissions (Fig. 2). Interestingly, all strains were obtained from patients on the neonatal intensive care unit (NICU). All patients/isolates were connected by at least one potential transmission event, thereby forming one cluster of patients (Fig. 2).

WGS and subsequent core genome-based SNP analysis were performed on the isolates and revealed one SNP cluster (“A”) when a cutoff value of 18 SNPs was applied (28). SNP cluster A included 10 of the isolates and 2 genetically distinct singletons (Fig. 2; Fig. S1). Thus, 13 of 17 potential transmissions (76.5%) were confirmed by SNP analysis.

The strains were also subjected to FTIR spectroscopy for typing. Isolates were considered to be clonal if their spectral similarity exceeded 75% as previously described for *K. pneumoniae* (11). This typing technique identified two clusters (FTIR clusters “1” and “2”) of isolates as well as two singletons (Fig. 2). All isolates in FTIR clusters 1 and 2 belonged to SNP cluster A, but the clonality of the isolates J00992 and J00924 and the other isolates in this SNP cluster was not recognized by FTIR spectroscopy. Thus, three of the confirmed transmission events were missed (Fig. 2). Isolates J00803 and J00645 were correctly identified as genetically distinct from all other isolates. No transmission events were falsely assumed by FTIR spectroscopy, resulting in high specificity (100%) and good to moderate sensitivity (77%) when compared to WGS analysis.

As the FTIR spectra are influenced to a large extent by the polysaccharides and capsule of the bacteria, the capsule types were extracted from WGS data using the online tool Kaptive (27). All isolates within SNP cluster A belonged to the same capsule type, so the discrepancy in phenotypic clustering by FTIR could not be explained by the genetic capsule type (Fig. S1).

Overall, the transmission surveillance showed high efficiency for *K. pneumoniae,* with 13 out of 17 potential transmissions confirmed by WGS (Fig. 2). Room contact seems to play an important role in the transmission of these pathogens.

### Transmission surveillance and typing of *E. cloacae* complex

**Fig 3:**
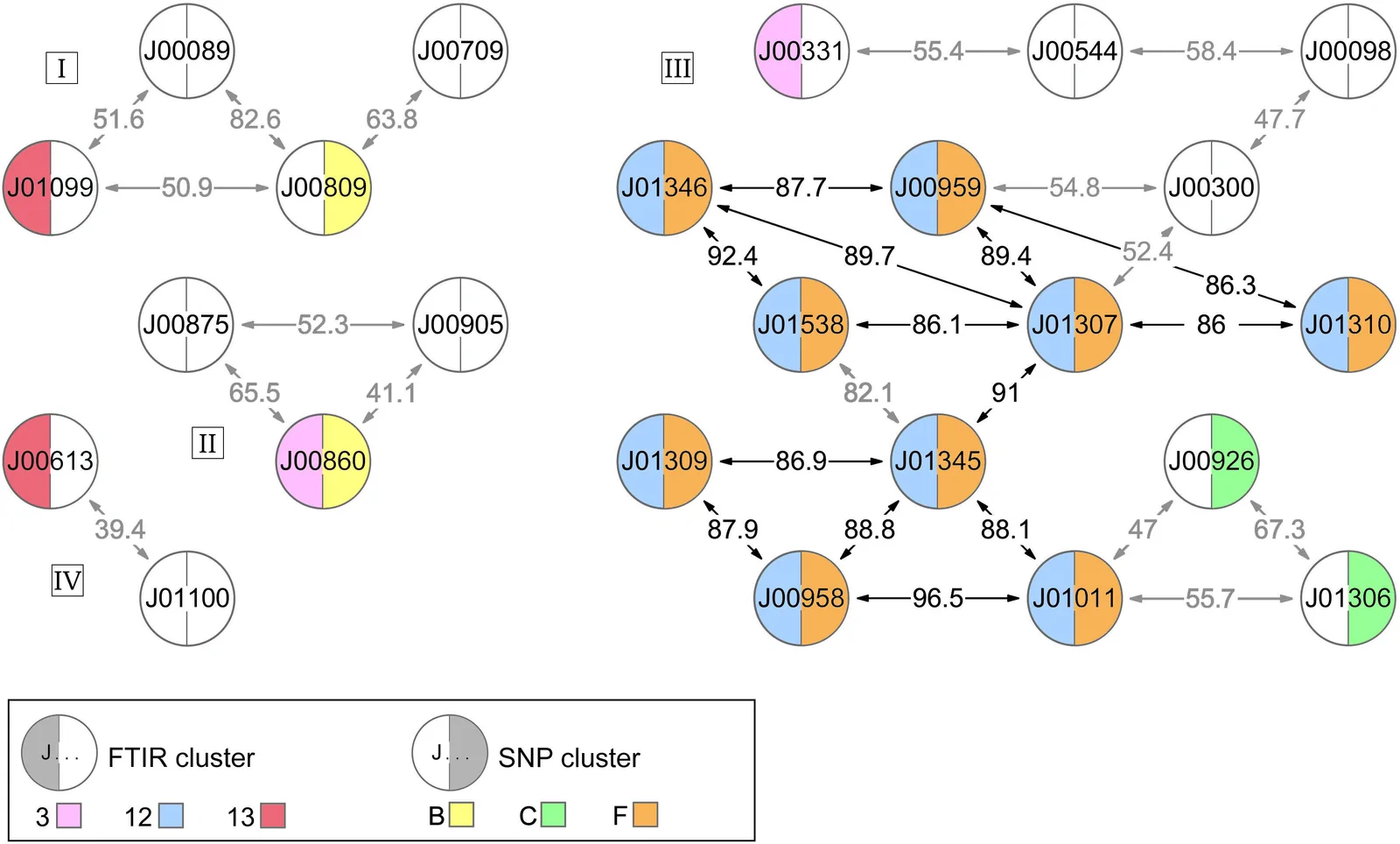
Transmission surveillance of *E. cloacae* complex. For *E. cloacae* complex, 30 potential transmissions (black and gray arrows) among 24 patients/isolates (circles with isolate ID) in 4 clusters (Roman numerals) were suspected based on epidemiological data. WGS revealed 3 SNP clusters (colored right half of the circles; cutoff value: 6 SNPs) and 11 singletons (uncolored). FTIR typing revealed 3 FTIR clusters (colored left half of the circles) and 11 singletons (uncolored). Arrows and numbers indicate the FTIR spectrum similarities between isolates above (black) and below (gray) the cutoff value (83%).

In total, 24 of 118 collected *E. cloacae* complex isolates were involved in 30 potential transmissions based on screening of hospital occupancy data. These putative transmissions could be grouped into four patient clusters (“I” through “IV,” Fig. 3) comprising between 2 and 15 patients. More than half of the patients from whom the isolates were retrieved were located in the NICU, forming one large patient cluster (cluster “III”). To further investigate the suspected transmissions, the isolates were subsequently subjected to typing by FTIR spectroscopy and WGS.

Core-genome SNP analysis revealed three genomic clusters (“B,” “C,” and “F”) when a cutoff value of 6 SNPs was applied, as well as 11 genetically distinct singletons (Fig. 3). Clusters “C” and “F” comprised nine and two patients, respectively, all of whom were part of patient cluster “III.” Thus, 13 of 22 suspected transmissions in this cluster could be confirmed. None of the presumed transmissions in clusters “I,” “II,” and “IV” were corroborated by SNP analysis. Interestingly, isolates J00809 and J00860 were clonally related (SNP cluster “B”) but were not linked by the epidemiological definition of suspected transmission used in this study (Fig. 3; Fig. S2). Inspection of occupancy data revealed that both patients never shared the same room but were treated at the same time on the same ward.

Similarity clustering of the isolate spectra obtained by FTIR spectroscopy revealed three clusters (“3,” “12,” and “13”) when the previously described cutoff value of 83% was applied (12). These clusters contained 13 isolates, whereas 11 were distinct singletons (Fig. 3). FTIR cluster “12” was completely concordant with SNP cluster “F.” However, FTIR spectroscopy failed to detect the clonality of isolates J00926 and J01306 (SNP cluster “C”) and isolates J00809 and J00860 (SNP cluster “B”). Additionally, the technique grouped isolates J01099 and J00613 as well as J00860 and J00331, which had no genetic or epidemiological link (Fig. 3). Typing by FTIR spectroscopy recognized 12 of 13 confirmed transmission events, resulting in a sensitivity of 95% and a specificity of 100%. The link between isolates J01538 and J01345 was the only one not detected because the spectral similarity between the two isolates was slightly below the cutoff value. All other suspected transmissions that were rejected by SNP analysis were also considered as “no transmission” by FTIR spectroscopy.

Taken together, transmission surveillance solely based on epidemiological data overestimated the number of actual transmissions confirmed by WGS. As for *K. pneumoniae*, room contact also seems to play an important role in the transmission of *E. cloacae* complex.

### Transmission surveillance and typing of *P. aeruginosa*

**Fig 4:**
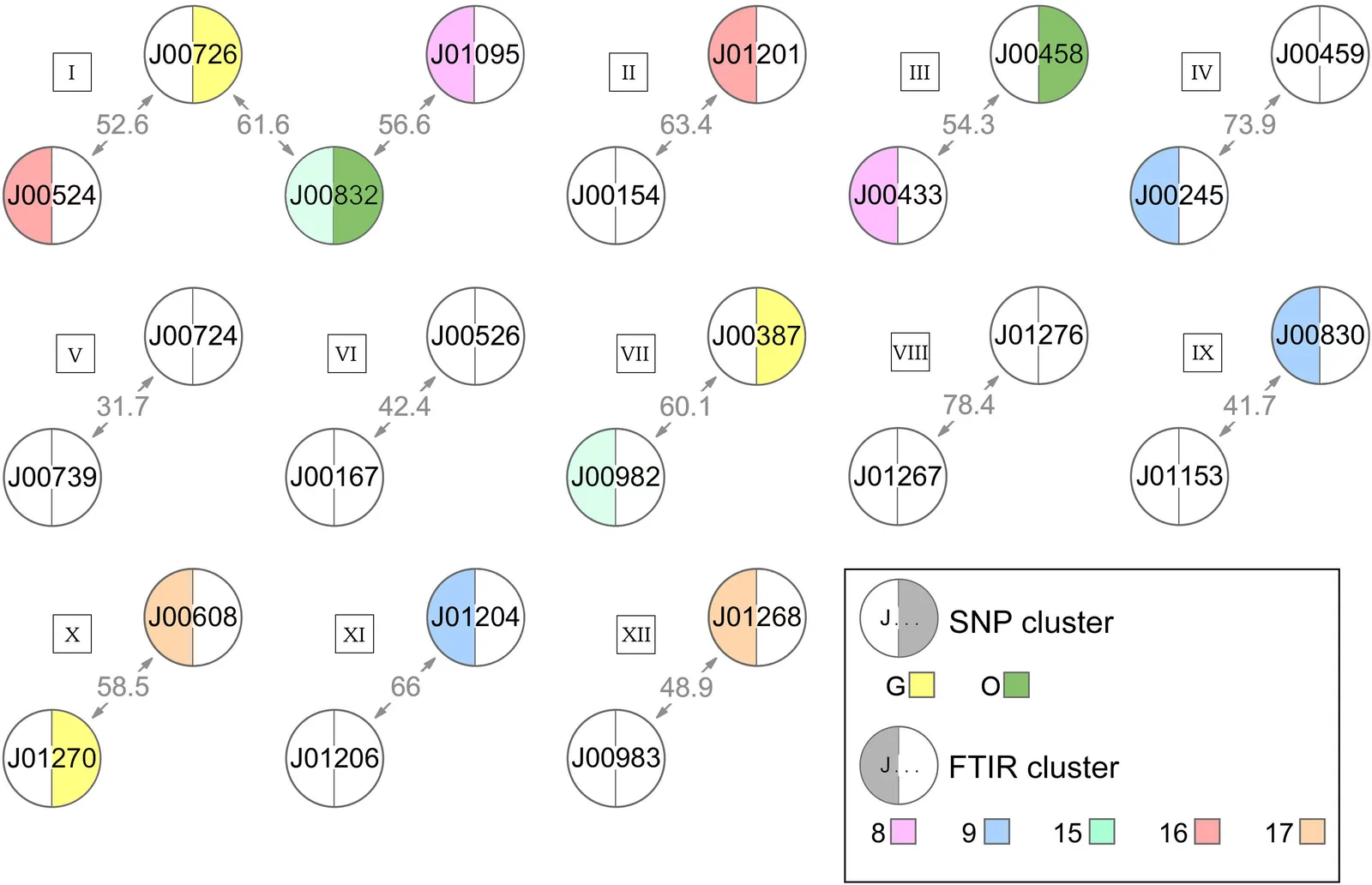
Transmission surveillance of *P. aeruginosa*. For *P. aeruginosa,* 14 potential transmissions (black and gray arrows) among 26 patients/isolates (circles with isolate ID) in 12 clusters (Roman numerals) were suspected based on epidemiological data. WGS revealed 2 SNP clusters (colored right half of the circles; cutoff value: 60 SNPs) and 21 singletons (uncolored). FTIR typing revealed 5 FTIR clusters (colored left half of the circles) and 15 singletons (uncolored). Arrows and numbers indicate the FTIR spectrum similarities between isolates above (black) and below (gray) the cutoff value (85%).

Screening of hospital occupancy data revealed 14 potential transmissions of *P. aeruginosa* involving 26 patients and their respective isolates. Most of the potential transmissions were suspected between pairs of only two patients, whereas only one cluster consisted of four patients (cluster “I”) (Fig. 4).

WGS and SNP typing of the isolates revealed a very heterogeneous strain collection. Moreover, when MLST was performed from the assembled genome sequences, only ST-253 and ST-395 were represented by more than one isolate. When choosing a cutoff value of 60 SNPs, isolates J00726, J00387, and J01270, as well as J00832 and J00458, formed SNP clusters G and O, respectively. Those clusters could be further separated into singletons by lowering the cutoff value to the previously described cutoff of 37 SNPs (Fig. S3) (28). Considering the high genetic mobility and mutation rate of *Pseudomonas aeruginosa,* the cutoff value of 60 SNPs was chosen for further analysis. Isolates within SNP clusters G and O showed no epidemiological link based on hospital occupancy data (Fig. 4). Furthermore, no presumed transmission event was verified by SNP typing.

FTIR spectroscopy, when using an 85% similarity threshold for clustering, revealed five groups of isolates (“8,” “9,” “15,” “16,” and “17”), but none of these strains were part of a suspected transmission event. Comparable to SNP typing, FTIR spectroscopy rightfully rejected all suspected transmissions, resulting in a specificity of 100% (Fig. 4).

Overall, patient-to-patient transmission of *P. aeruginosa* seems to be a rare event since epidemiological screening strongly overestimated the number of transmissions in our setting. FTIR spectroscopy struggled with the diverse strain distribution in this setting, which resulted in occasional clustering of isolates that were genetically distinct.

### Transmission surveillance and typing of *S. aureus*

**Fig 5:**
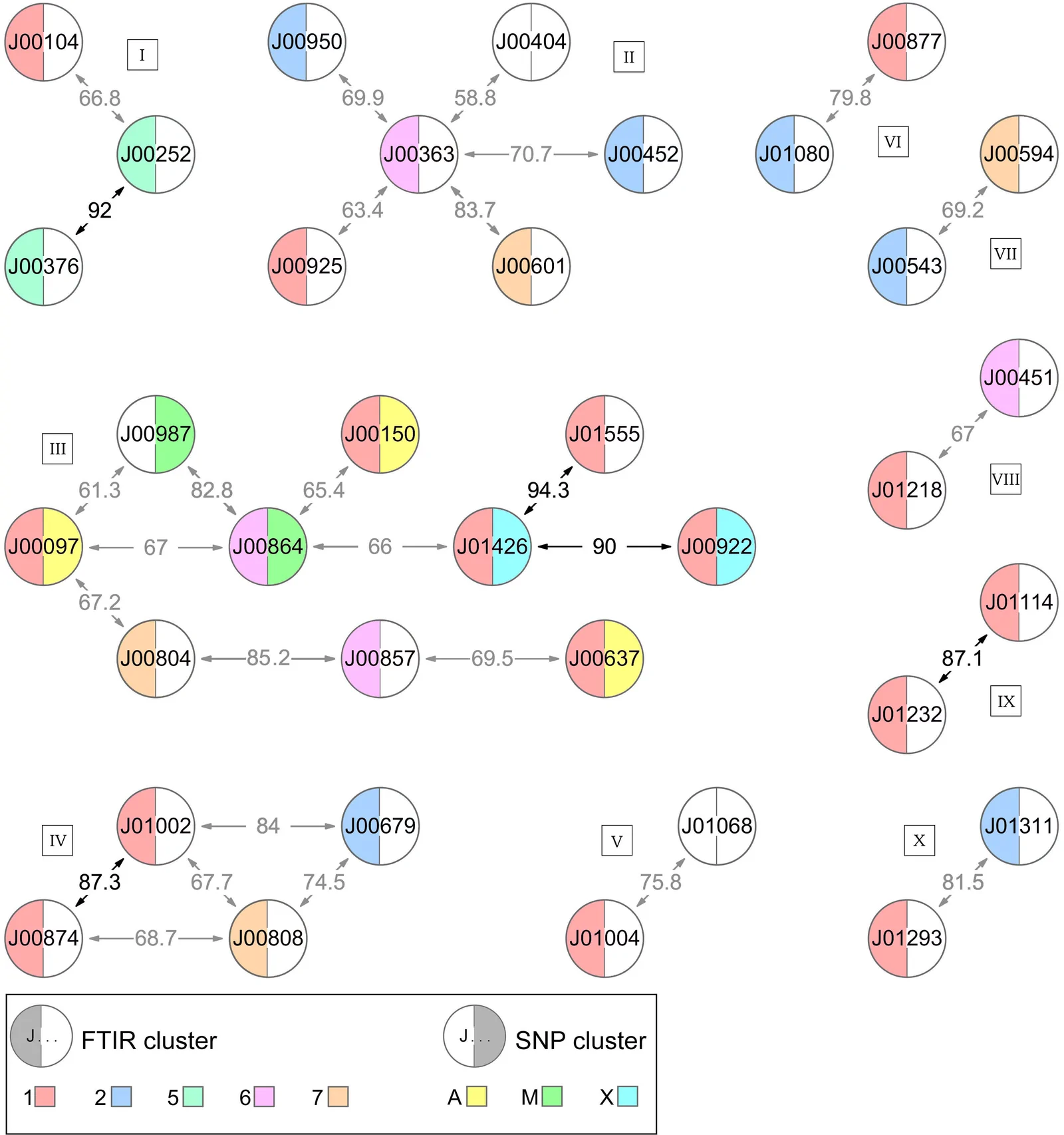
Transmission surveillance of *S. aureus*. For *S. aureus,* 28 potential transmissions (black and gray arrows) among 35 patients/isolates (circles with ID) in 10 clusters (Roman numerals) were suspected based on epidemiological data. WGS revealed 3 SNP clusters (colored right half of the circles; cutoff value: 15 SNPs) and 28 singletons (uncolored). FTIR typing revealed five FTIR clusters (colored left half of the circle) and three singletons (uncolored). Arrows and numbers indicate the FTIR spectrum similarities between isolates above (black) and below (gray) the cutoff value (87%).

Transmission surveillance for *S. aureus* detected 28 possible transmission events that could be grouped into 10 patient clusters (“I” through “X,” Fig. 5). These clusters contained 35 patients with their respective bacterial isolate. Most clusters contained only a pair of patients, whereas the largest cluster (“III”) consisted of 10 patients.

WGS analysis of the isolates revealed a genetically diverse population. In total, 18 *spa* types and 14 different sequence types were present in the collection (Fig. S4). SNP analysis grouped the isolates into 28 singletons and 3 clusters (“A,” “M,” and “X”) consisting of three (“A”) or two (“M” and “X”) isolates, based on a 15 SNP cut off adopted from previous literature (28). Two transmission events involving isolates from SNP clusters “M” and “X” could be confirmed, whereas all other suspected transmissions were rejected by the genetic analysis. Interestingly, three patients, from whom the isolates in SNP cluster “A” were found, belonged to the same patient cluster “III” but had no contact when the previously defined epidemiological criteria were applied (Fig. 5).

FTIR spectroscopy detected five clusters (“1,” “2,” “5,” “6,” and “7”) of isolates based on an 87% spectral similarity cut off. Four suspected transmission events involved isolates that showed a high spectral similarity but were genetically unrelated. Only one pair of isolates (J01426 and J00922) was found to be involved in a transmission and exhibited genetic and spectral relatedness. Overall, from 28 suspected transmissions, only 2 were genetically confirmed, and only one of these was also detected by FTIR spectroscopy typing. Three clonally related strains (J000097, J00150, and J00637) that had no epidemiological link were grouped in the same cluster by FTIR analysis (Fig. 5).

Comparable to the results with *P. aeruginosa*, occupancy-based transmission surveillance starkly overestimated the number of confirmed transmissions of *S. aureus*. In addition, FTIR spectroscopy had difficulties in assessing a very heterogeneous *S. aureus* population, which resulted in occasional clustering of strains that were genetically distinct, yielding a sensitivity of 50% and specificity of 84%.

## DISCUSSION

In this study, the performance and feasibility of a hospital-wide pathogen transmission surveillance combining epidemiological information with strain typing results from WGS, as well as FTIR spectroscopy, was examined.

The efficiency of this elaborate transmission surveillance tool appeared highly species dependent. While for *S. aureus* and *P. aeruginosa* only very few transmissions could be confirmed, for *K. pneumoniae* and *E. cloacae* complex, the surveillance was very effective. The low hit rate for *S. aureus* and *P. aeruginosa* might be due to predominant transmission routes other than patient-to-patient transmission. For *P. aeruginosa,* the patient environment plays a key role in its spread within the hospital. The water drainage system, particularly sinks and siphons, has been found as a main reservoir and source in the spread of outbreak strains in a large number of *P. aeruginosa* outbreaks (29–31). To detect transmissions driven by the patient environment, it might be necessary to expand the criteria defining potential transmissions and include patients who were admitted to the same room sequentially but not simultaneously.

One major risk factor for the development of nosocomial *S. aureus* infection is the nasal colonization, as infections with *S. aureus* frequently occur due to endogenous spread (32, 33). In this study, transmissions were only suspected among methicillin-susceptible *S. aureus* (MSSA) isolates. Except for the NICU, there is no comprehensive routine screening for colonization with MSSA established, making it difficult to differentiate between endogenous and exogenous infections. The high genetic diversity of *S. aureus* strains in this study indicates a consecutive ingress of different strains rather than transmission of certain clones. All transmissions of MSSA involved patients within the NICU. Likewise, previous studies have examined the epidemiology and potential transmission routes of MSSA in NICUs, revealing several MSSA outbreaks (32, 34). Another study performed WGS for strain typing in a non-outbreak setting and revealed a series of clonal isolates suggesting nosocomial transmission (35).

For *K. pneumoniae* and *E. cloacae* complex, a high number of transmissions were detected by the demonstrated surveillance tool. Most of these transmissions occurred between patients in the NICU who were colonized with these pathogens, with no evidence of infection. The high number of transmissions within the NICU might be partly explained by the elaborate screening for susceptible as well as resistant isolates conducted in the NICU on a weekly basis. Increased screening in non-NICU wards might therefore also increase the detection rate of pathogen transmissions. The very long median length of stay in the NICU, and not least the vulnerability and susceptibility of NICU patients to colonization and infection with gram-negative rods, might contribute to these high transmission rates (36). *K. pneumoniae* and *E. cloacae* complex are frequently involved in outbreaks in NICUs (37, 38). The presented transmission surveillance might be a helpful instrument in early outbreak detection in these wards.

One limitation of the study is that potential transmissions were defined solely based on patients being co-located in the same room. While this approach may not capture all transmission events, including those occurring via environmental reservoirs such as sinks, siphons, or shared surfaces, it allowed us to implement a feasible, hospital-wide real-time surveillance system using routinely collected epidemiological data and isolates. Expanding the criteria to include patients on the same ward or incorporating additional environmental sampling could further increase the number of detected transmission events in future studies. As the hands of healthcare workers are a major vector of transmission, it may be reasonable to expand the criteria for transmission and focus not only on patients co-located in the same room but also on inpatients on the same ward. In our data, we do see evidence that transmissions occurred between patients on the same ward for *E. cloacae* complex (SNP cluster B) and *S. aureus* (SNP cluster A). If the criteria for potential transmission were expanded, we would expect to find even more transmissions that are overlooked when focusing only on patients co-located in the same room.

Even though referred to as patient-to-patient transmission, the actual sources and transmission pathways remain unrevealed in this setting. Presumable vectors, such as the hands of healthcare workers and patient environment, cannot be ruled out, as no additional screenings were conducted. If outbreaks were to be detected, further investigations would have to be implemented to identify the outbreak source.

Although early identification of emerging outbreaks is crucial to implement infection control measures on time, a series of recent studies and reviews have addressed the lack of standardized surveillance tools used by infection prevention and control teams (39, 40). Schröder et al. (41) developed an automated alarm system for nosocomial outbreaks based on the number of certain bacterial pathogens detected over time. When the number of detected pathogens exceeds a predefined baseline, an automated alarm is triggered, and outbreak investigation measures, including strain typing, are performed. Another approach was conducted by Mellmann et al. (42), who performed WGS for real-time strain typing of certain MDROs. Transmission was assumed if clonal isolates were obtained from patients located on the same ward within 1 month. Most existing transmission surveillance tools focus on the spread of multi-drug-resistant bacteria, risking a blind angle and missing the spread of antibiotic-susceptible bacteria (43). Therefore, in this study, isolates were included independent of their resistance pattern. If focusing on multi-drug-resistant isolates, all 32 transmissions would have been missed in this setting. The inclusion of susceptible isolates, of course, raises the number of potential transmissions and isolates subjected to typing significantly. To avoid excessive expenses for WGS, FTIR might be a useful, fast, and economical strain typing tool.

The accuracy of FTIR as a strain typing method was assessed compared to the gold standard WGS in the context of this transmission surveillance. The performance of FTIR typing compared to WGS was highly species dependent. FTIR has already been described as a promising tool for typing of *K. pneumoniae* and *E. cloacae* complex (11, 12). We found high concordance of FTIR and WGS for typing of *K. pneumoniae*, but two isolates were inexplicably misclassified by FTIR. Although the spectra are largely influenced by the polysaccharides and capsule of the bacteria, the genetic capsule types could be ruled out as the cause for this phenotypic discrepancy.

Both methods showed high congruency for typing of *E. cloacae* complex, with only one transmission missed by FTIR typing. Species and subspecies within the *E. cloacae* complex can be poorly differentiated by MALDI-TOF. Improving the discriminatory power of MALDI-TOF of species within the *E. cloacae* complex would avoid a number of redundant suspected transmissions and unnecessary typing procedures.

FTIR showed very low discriminatory power for typing *S. aureus,* clustering genetically distinct isolates in one FTIR cluster and thereby overestimating the number of transmissions. A previous study demonstrated the capability of FTIR to distinguish capsule types of *S. aureus* but not between different clonal complexes of *S. aureus* (44). In a recent study, difficulties regarding the reproducibility of FTIR spectra were reported, and therefore, the discriminatory power of FTIR compared to WGS could not be determined (45).

Although FTIR rightfully rejected all epidemiologically suspected transmissions of *P. aeruginosa*, when comparing all isolates, we found low concordance of clustering by FTIR and WGS. In a previous study, FTIR was successfully applied for typing *P. aeruginosa* and could accurately distinguish different STs (16). In the above-mentioned study, *P. aeruginosa* isolates were retrieved from outbreaks, while in our setting, isolates were genetically very distinct. Further studies are needed to determine the discriminatory power of FTIR typing of *P. aeruginosa* compared to WGS in an outbreak setting.

Overall, we present a useful pathogen transmission surveillance combining epidemiological information and strain typing results. Adjustments regarding transmission criteria, wards, and species included in the surveillance might be required to increase the efficiency of the surveillance tool. For high-throughput analysis, FTIR is a powerful, fast, and low-cost alternative to WGS, but as the method is highly species dependent, pathogens subjected to FTIR typing should be chosen thoroughly.

## Data Availability

The genome sequences used in this study are publicly available in the European Nucleotide Archive (ENA) under the accession number PRJEB62643.

## SUPPLEMENTAL MATERIAL

Supplemental figures spectrum.02230-25-s0001.docx Fig. S1 to S4.

ASM does not own the copyrights to Supplemental Material that may be linked to, or accessed through, an article. The authors have granted ASM a non-exclusive, world-wide license to publish the Supplemental Material files. Please contact the corresponding author directly for reuse.
