# Supplementary material for: Hospital-wide pathogen transmission surveillance combining epidemiological, genetic, and spectroscopic approaches
Source: Microbiol Spectr. 2026 May 26;14(7):e02230-25. doi: 10.1128/spectrum.02230-25 (PMC13339811; doi:10.1128/spectrum.02230-25)
Supplement: Supplemental figures — Fig. S1 to S4. [file spectrum.02230-25-s0001.docx]

**SUPPLEMENTARY MATERIAL**

**A**


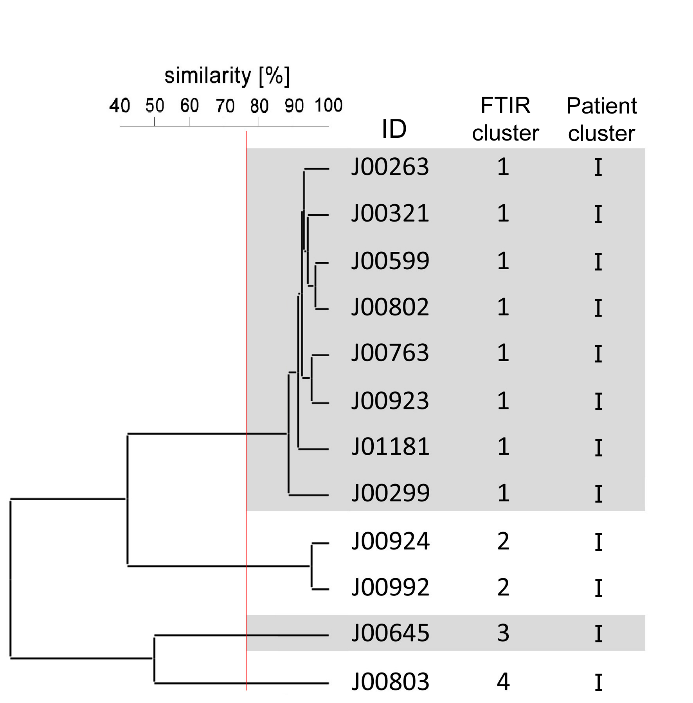


**B**


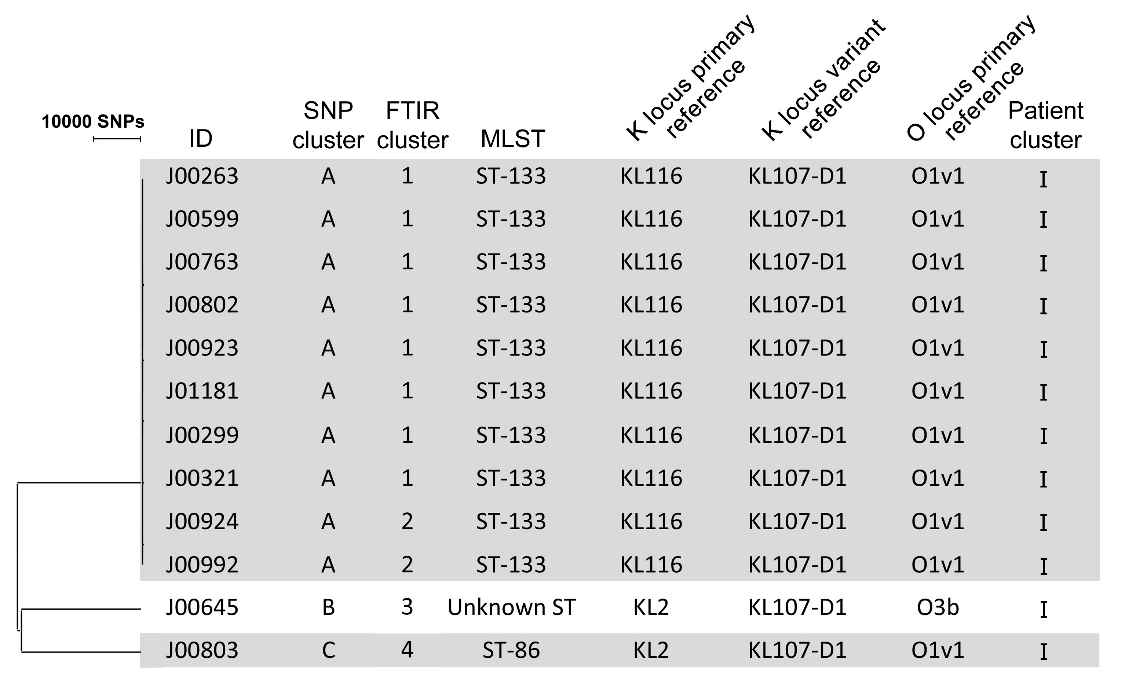


**Figure S1 Spectral and genomic clustering of *Klebsiella pneumoniae* isolates involved in potential transmissions**. (A) FTIR clustering of *K. pneumoniae* isolates. Similarity cut-off value [75%] for FTIR spectrum clustering is shown as a vertical red line. **(B)** SNP-based phylogeny of *K. pneumoniae* isolates. The multi-locus sequence type (MLST) was extracted from the assembled genome sequences. Capsule types were determined using the online tool Kaptive. FTIR clustering was derived from panel **(A)**.

**A**


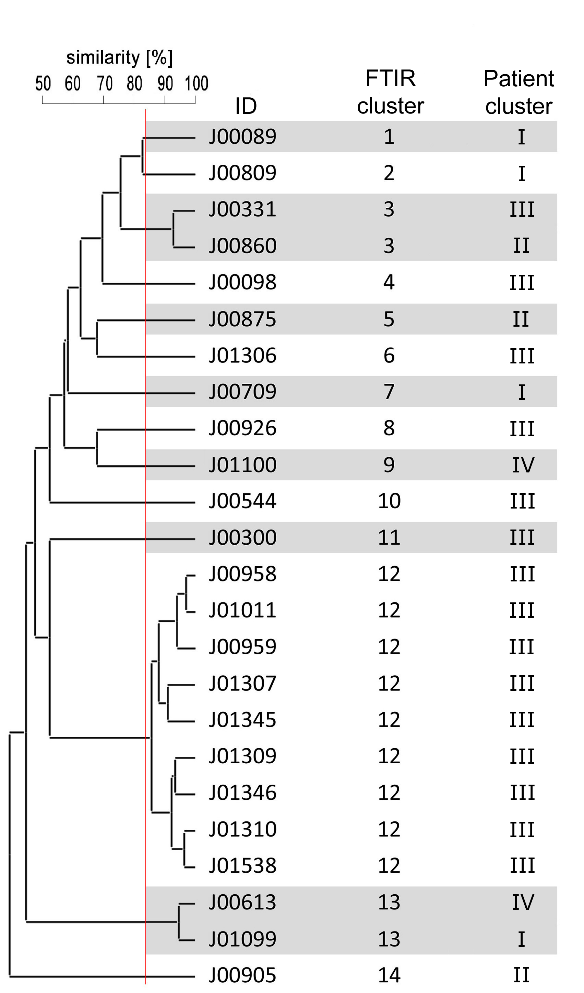


**B**


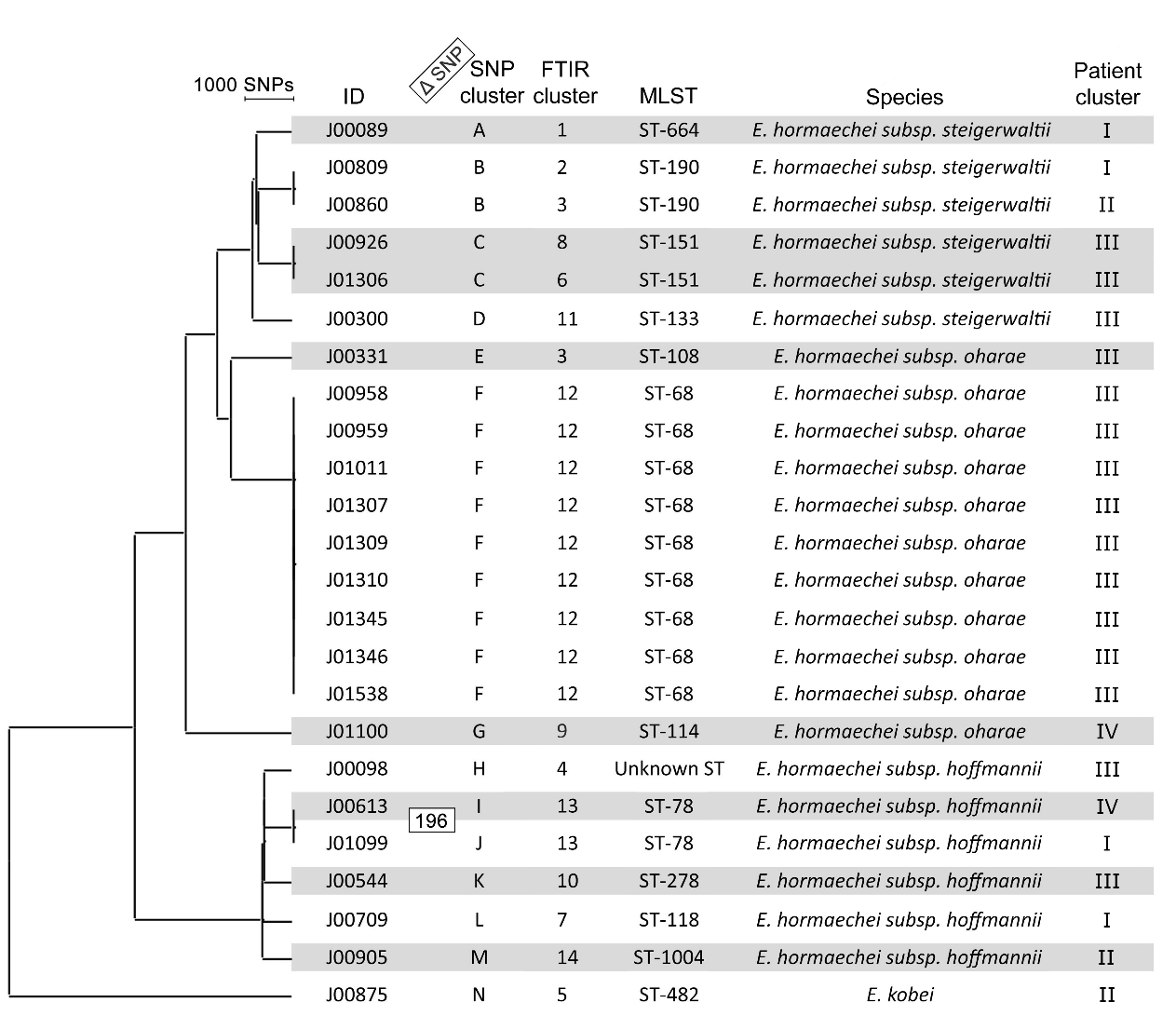


**Figure S2 Spectral and genomic clustering of *Enterobacter cloacae complex* isolates involved in potential transmissions.** **(A)** FTIR clustering of *E. cloacae complex* isolates. Similarity cut-off value [83%] for FTIR spectrum clustering is shown as a vertical red line. **(B)** SNP-based phylogeny of *E. cloacae complex* isolates. The multi-locus sequence type (MLST) was extracted from the assembled genome sequences. Species and subspecies identification was performed calculating the average nucleotide identity compared to the reference genome. The number of SNPS between isolates belonging to the same MLST is marked by a rectangle [∆ SNP]. FTIR clustering was derived from panel **(A)**.

**A B**


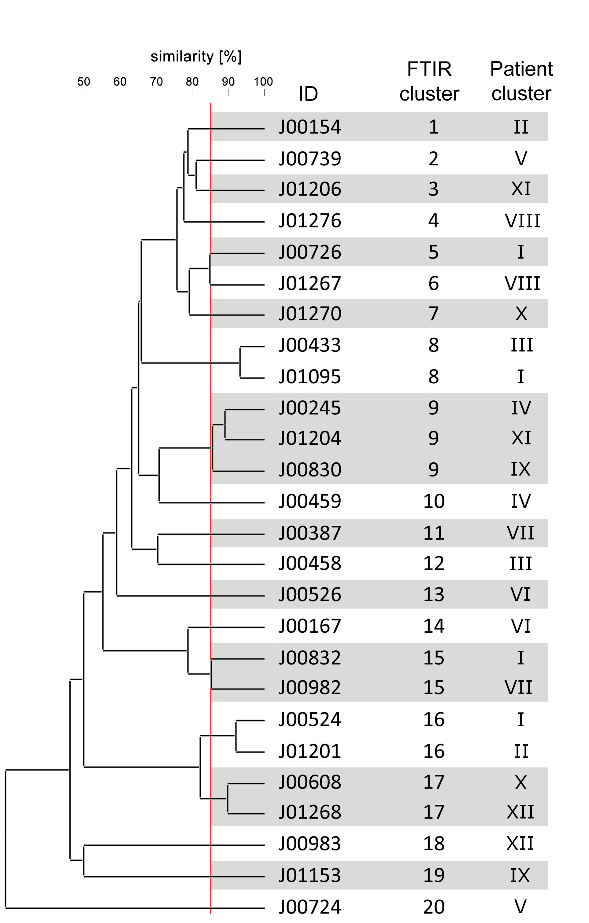

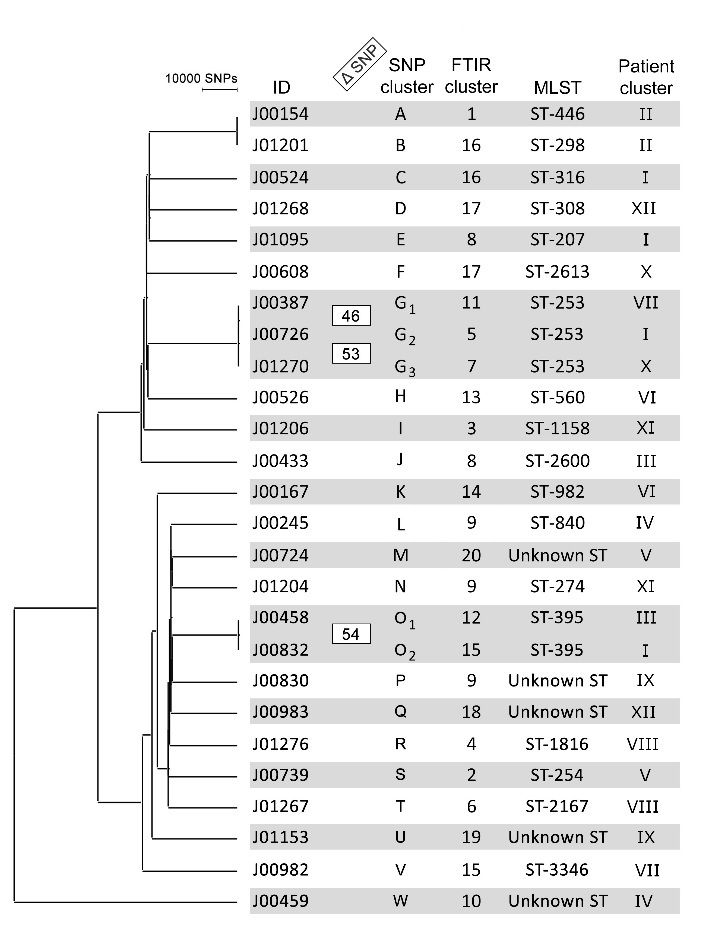


**Figure S3 Genomic and spectral clustering of *Pseudomonas aeruginosa* isolates involved in potential transmissions.** **(A)** FTIR clustering of *P. aeruginosa* isolates. Similarity cut-off value [85%] for FTIR spectrum clustering is shown as a vertical red line. **(B)** SNP-based phylogeny of *P. aeruginosa* isolates. The multi-locus sequence type (MLST) was extracted from the assembled genome sequences.

When choosing 60 SNPs as cut-off value two clusters (G and O) were formed. Those clusters were divided into singletons when choosing a lower cut-off value of 37 SNPs. The number of SNPS between isolates belonging to the same MLST is marked by a rectangle [∆ SNP]. FTIR clustering is derived from panel **(A)**.

**A B**


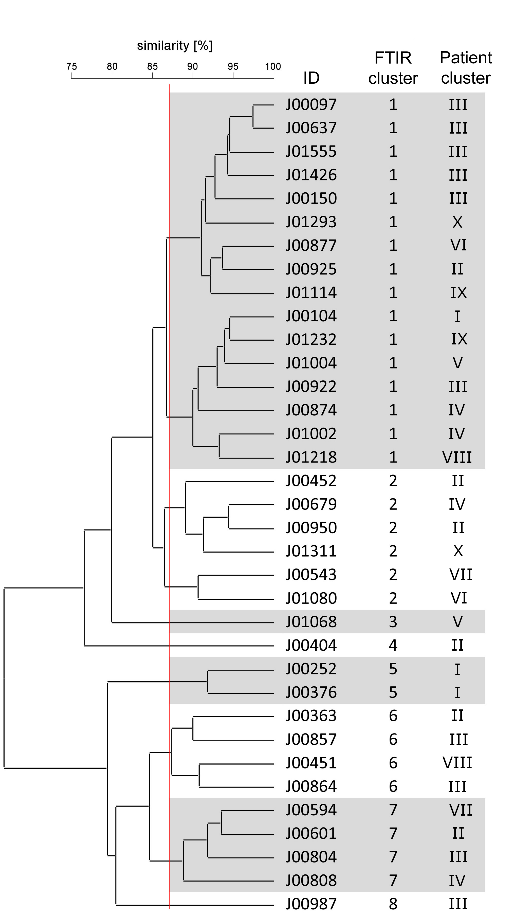
­
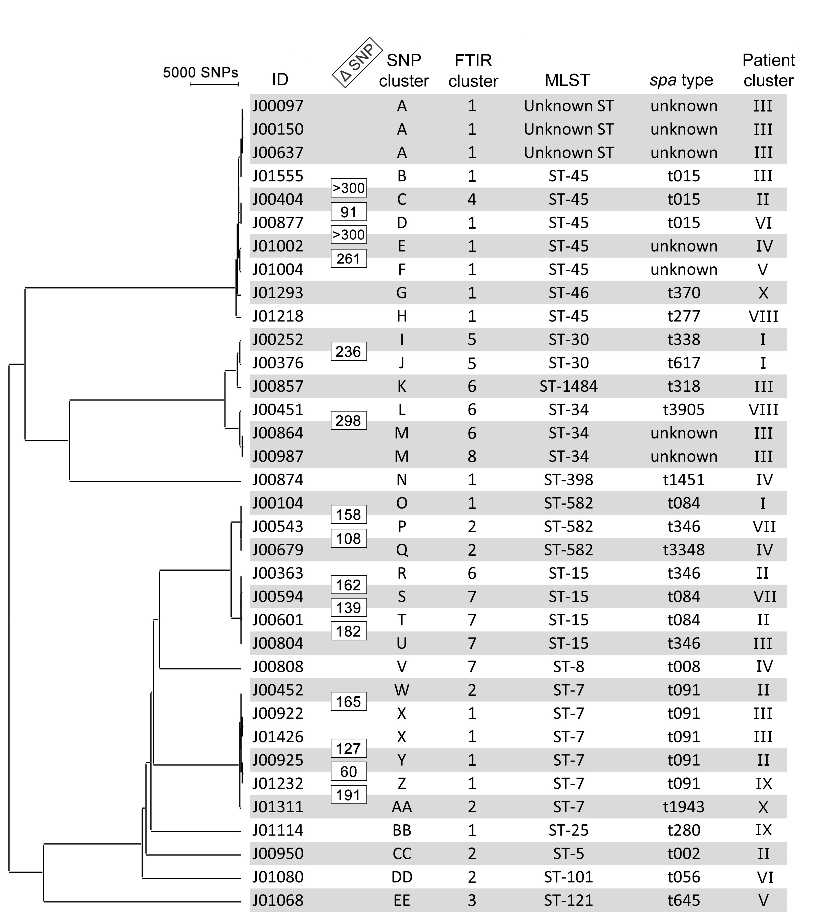


**Figure S4 Genomic and spectral clustering of *Staphylococcus aureus* isolates involved in potential transmissions.** **(A)** FTIR clustering of *S. aureus* isolates. Similarity cut-off value [87%] for FTIR spectrum clustering is shown as a vertical red line. **(B)** SNP-based phylogeny of *S. aureus* isolates. The multi-locus sequence type (MLST) was extracted from the assembled genome sequences. The number of SNPS between isolates belonging to the same MLST is marked by a rectangle [∆ SNP]. FTIR clustering is derived from panel **(A)**.
